# Structure and dynamics of the staphylococcal pyridoxal 5-phosphate synthase complex reveal transient interactions at the enzyme interface

**DOI:** 10.1016/j.jbc.2024.107404

**Published:** 2024-05-21

**Authors:** Angélica Luana C. Barra, Najeeb Ullah, Hévila Brognaro, Raissa F. Gutierrez, Carsten Wrenger, Christian Betzel, Alessandro S. Nascimento

**Affiliations:** 1 São Carlos Institute of Physics, University of São Paulo, São Carlos, Brazil; 2 Institute of Biochemistry and Molecular Biology, Laboratory for Structural Biology of Infection and Inflammation, University of Hamburg, Hamburg, Germany; 3 Department of Biochemistry, Bahauddin Zakariya University, Multan, Pakistan; 4 Unit for Drug Discovery, Department of Parasitology, Institute of Biomedical Sciences, University of São Paulo, São Paulo, Brazil

**Keywords:** *Staphylococcus aureus*, vitamin B6, PLP synthase, protein crystallization, oligomeric state

## Abstract

Infectious diseases are a significant cause of death, and recent studies estimate that common bacterial infectious diseases were responsible for 13.6% of all global deaths in 2019. Among the most significant bacterial pathogens is *Staphylococcus aureus*, accounting for more than 1.1 million deaths worldwide in 2019. Vitamin biosynthesis has been proposed as a promising target for antibacterial therapy. Here, we investigated the biochemical, structural, and dynamic properties of the enzyme complex responsible for vitamin B6 (pyridoxal 5-phosphate, PLP) biosynthesis in *S. aureus*, which comprises enzymes *Sa*Pdx1 and *Sa*Pdx2. The crystal structure of the 24-mer complex of *Sa*Pdx1-*Sa*Pdx2 enzymes indicated that the *S. aureus* PLP synthase complex forms a highly dynamic assembly with transient interaction between the enzymes. Solution scattering data indicated that *Sa*Pdx2 typically binds to *Sa*Pdx1 at a substoichiometric ratio. We propose a structure-based view of the PLP synthesis mechanism initiated with the assembly of *Sa*PLP synthase complex that proceeds in a highly dynamic interaction between Pdx1 and Pdx2. This interface interaction can be further explored as a potentially druggable site for the design of new antibiotics.

*Staphylococcusaureus* is an opportunistic pathogen that colonizes the nasal mucosa in 20 to 40% of the general population (1). Despite its widespread presence in human mucosa, *S. aureus* infections raise concerns for human health. Ikuta and coworkers recently estimated approximately 13.7 million infection-related deaths worldwide in 2019 (2). Among the pathogens investigated, *S. aureus* was the top-ranked bacterial pathogen, associated with more than 1 million deaths in 2019, corresponding to 40 million years of life lost. If considered a cause of death, *S. aureus* would rank as the top 15th cause of death in 2019 worldwide (2). Note that these estimates were focused only on bacterial infections and left out the analysis of infections caused by *Mycobaterium tuberculosis* (2).

According to the Network of Healthcare Safety in the USA, *S. aureus* is the second most frequent pathogen in health care-associated infections, after *Escherichia coli*, and the top ranked pathogen associated with surgical site infections (3). Worryingly and alarmingly, the same report showed that 40 to 79% of *S. aureus* isolates were resistant to methicillin (3). The spread of resistance against beta-lactam antibiotics such as methicillin suggests a scenario where the pharmacological options for the treatment of these infections will be very limited or even nonexistent. Also, a great concern about drug-resistant *S. aureus* is infection outbreaks in hospitals, which increase the risk for even ordinary medical procedures such as surgeries, organ transplants, hemodialysis, and chemotherapy (1, 4, 5, 6, 7, 8). During the coronavirus disease pandemic, this problem has become even more evident. Many secondary infections in coronavirus disease patients admitted to the intensive care unit are caused by methicillin-susceptible and methicillin-resistant *S. aureus* strains (9, 10, 11, 12). Furthermore, bacterial coinfection was associated with increased mortality in coronavirus disease (11).

Walsh and Wencewicz noted that the existing antibiotics are limited to only five clinically validated antibacterial targets/pathways: (*i*) the inhibition of cell wall biosynthesis; (*ii*) the inhibition of protein synthesis; (*iii*) the inhibition of DNA or RNA synthesis; (*iv*) the inhibition of folate biosynthesis, which damages DNA synthesis; and (*v*) the disruption of membrane integrity (13). Consequently, it is urgent to identify novel molecular targets for antimicrobial drug discovery investigations.

*De novo* biosynthesis pathways for vitamins have been proposed as interesting potential targets for the development of new antimicrobials (14, 15, 16, 17). The vitamin B6 biosynthetic pathway, in particular, is conserved in most bacteria, fungi, and plants but not in mammals, making it interesting in terms of specificity and reduced side effects. A multimeric assembly of two enzymes, Pdx1 and Pdx2, synthesizes pyridoxal 5-phosphate (PLP), the active form of vitamin B6, as well as its vitamers. Pdx2 has glutaminase activity, converting L-glutamine into L-glutamate and an ammonia molecule. The latter is delivered to Pdx1, which synthesizes PLP using ammonia, ribose 5-phosphate (R5P), and glyceraldehyde 3-phosphate (G3P) (18).

From a structural perspective, to date, only four three-dimensional structures of the PLP synthase have been reported: the complexes from *Bacillus subtilis* (PDB ID 2NV2) (19), *Thermotoga maritima* (PDB ID 2ISS) (20), *Plasmodium berghei/falciparum* (PDB ID 4ADS) (21), and *Geobacillus stearothermophilus* (PDB ID 4WXY) (22). These structural investigations also showed that Pdx1 can be observed in different oligomeric species in solution. For example, *Saccharomyces cerevisiae* Pdx1 was found to be hexameric (23), while most of the structurally characterized Pdx1 enzymes were reported to be dodecameric. *Bacillus subtilis* Pdx1, on the other hand, was found to form both hexamers and dodecamers in solution, as investigated by analytical ultracentrifugation (19). It has also been observed that adding substrate or a mutant Pdx2 shifts the Pdx1 equilibrium toward the dodecamer formation (19, 21), suggesting that changes in the environment could alter the equilibrium among the species.

Here, we provide, for the first time, experimental evidence for the dynamic assembly of the PLP synthase complex combining data obtained from X-ray small angles using solution scattering and from crystal structures, as well as complementary biophysical and biochemical analyses. Overall, our study provides the first insights into the native PLP synthase assembly mechanism, which is exploitable for future drug discovery.

## Results

### *Sa*Pdx2 enhances PLP synthesis by *Sa*Pdx1

**Figure 1:**
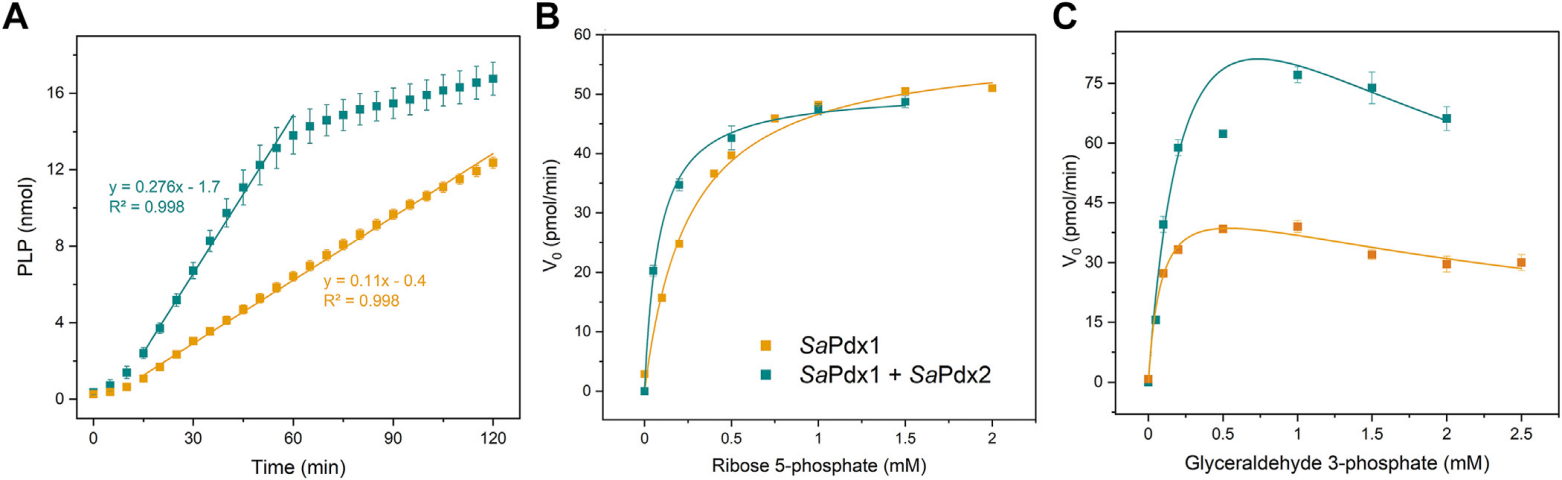
**PLP synthesis by *Sa*Pdx1 and *Sa*PLP synthase.***A*, the Schiff base concentration over time is increased for *Sa*PLP synthase (*Sa*Pdx1 + *Sa*Pdx2, *cyan curve*) compared to *Sa*Pdx1 and ammonia (*orange curve*). The linear fit of the concentration over time shows an increase of 2.5 times favoring *Sa*PLP synthase. *B* and *C*, michaelis‒Menten kinetics as a function of R5P (*B*) and G3P (*C*). The obtained kinetic parameters are shown in Table 1. These results were obtained from three independent experiments, each performed in triplicate. G3P, glyceraldehyde 3-phosphate; PLP, pyridoxal 5-phosphate; R5P, ribose 5-phosphate.

*Sa*Pdx1 and *Sa*Pdx2 were expressed in *E. coli* and purified to homogeneity (Fig. S1). The synthesis of PLP was monitored over time by the formation of Schiff’s base, as previously described (20). As expected, *Sa*Pdx1 can synthesize PLP when ammonia, G3P, and R5P are available in the medium. However, the synthesis efficiency is increased by a factor of 2.5 when Pdx2 and glutamine are added to the medium instead of ammonia (Fig. 1).

The Michaelis‒Menten kinetics monitored as a function of the R5P concentration (Fig. 1*B*) showed an increase in the apparent *k*_*cat*_/K_M_ ratio of approximately 21 times for the *Staphylococcus aureus PLP (Sa*PLP) complex compared to *Sa*Pdx1 + ammonia, mostly due to a decrease in apparent K_M_ (Table 1). When the G3P concentration was varied (Fig. 1*C*), the apparent *k*_*cat*_/K_M_ ratio increased 5.7 times, with approximately an 18-fold increase in the apparent *k*_*cat*_ favoring the *Sa*PLP complex (Fig. 1 and Table 1). Interestingly, we observe that *Sa*Pdx1 is inhibited at higher concentrations of G3P (Fig. 1*C*). This inhibition was not previously known, as far as we are aware, and must be due to the higher concentrations of G3P used in the assay. On the other hand, the high values found for K_i_ (1.9 and 3.3 mM for *Sa*PLP and *Sa*Pdx1, respectively) probably rule out an effective role in enzyme inhibition *in vivo*.

**Table 1:** Kinetic parameters for SaPdx1 and SaPLP synthase complex

Complex	Specific activity (pmol min^−1^ mg^−1^)	Ribose 5-phosphate		Glyceraldehyde 3-phosphate		
		K_M_ (mM)	*k*_*cat*_/K_M_ (min^−1^ mM^−1^)	K_M_ (mM)	*k*_*cat*_/K_M_ (min^−1^ mM^−1^)	K_i_ (mM)
*Sa*PLP Synthase	1125 ± 70	0.08 ± 0.01	0.5 ± 0.1	0.28 ± 0.09	0.4 ± 0.1	1.9 ± 0.9
*Sa*Pdx1 + ammonia	465 ± 20	0.26 ± 0.03	0.024 ± 0.006	0.09 ± 0.03	0.07 ± 0.03	3.3 ± 0.9

These results were obtained from three independent experiments, each performed in triplicate.

SaPLP, *Staphylococcus aureus* pyridoxal 5-phosphate.

Taken together, these results indicate that the *Sa*PLP complex delivers ammonia more efficiently to *Sa*Pdx1 than the diffusion of ammonia in the medium.

### The chemical environment can cause a shift in the equilibrium of *Sa*Pdx1 species in solution

The *Sa*Pdx1 multimeric assembly was assessed by applying dynamic light scattering as well as size-exclusion chromatography (SEC). We observed that when purified in Tris–HCl buffer, *Sa*Pdx1 preferentially formed hexamers in solution. On the other hand, when purified in sulfate or phosphate buffers, the enzyme preferentially formed dodecamers, the proposed active form of *Sa*Pdx1 in the PLP complex (Fig. S2), suggesting that the chemical environment can affect the equilibrium among *Sa*Pdx1 species in solution.

**Figure 2:**
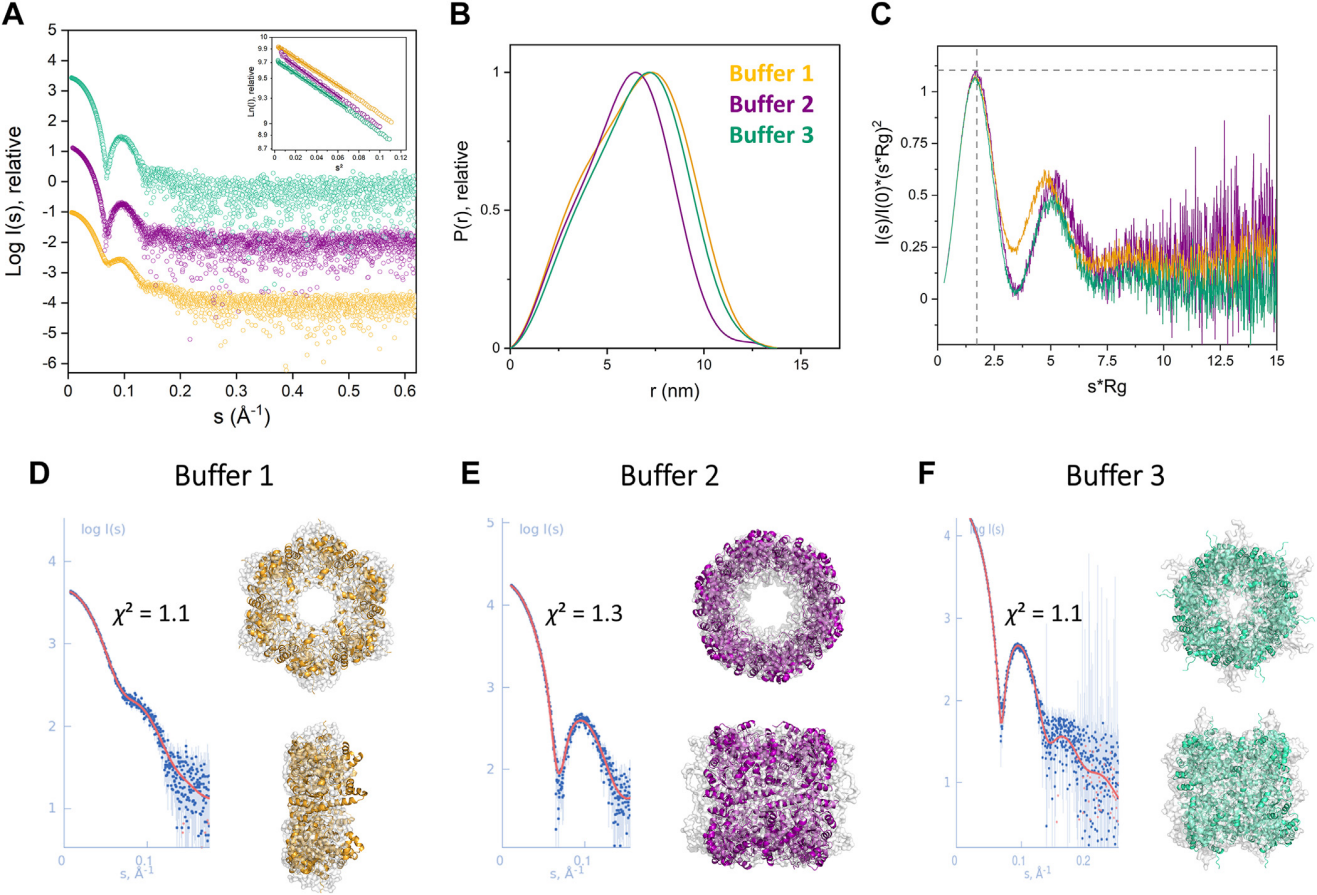
**SAXS data of *Sa*Pdx1 obtained in batch (buffer 1) or SEC-SAXS (buffers 2 and 3) measurements.** Different buffer conditions are *colored in yellow* (buffer 1: 50 mM Tris–HCl pH 8, 150 mM NaCl), *purple* (buffer 2: 50 mM Tris–HCl pH 8, 200 mM Na_2_SO_4_), and *green* (buffer 3: 100 mM Na_2_HPO_4_ pH 8, 150 mM NaCl). *A*, solution scattering X-ray intensity pattern in relative intensity units with their respective Guinier plots (inset) (*B*) p(r) functions in relative scale. *C*, dimensionless *Kratky plots* with the globular protein reference as the *dotted line*. *Ab initio* GASBOR models in orthogonal views, their fits (χ^2^) with the experimental data, and superimposed with the crystallographic structure of SaPdx1 are shown in *cartoon representation* (*D*) in buffer 1, (*E*) buffer 2, and (*F*) buffer 3. SAXS, small-angle X-ray scattering; SEC, size-exclusion chromatography.

The species identified with SEC were further analyzed by applying small-angle X-ray scattering (SAXS) experiments. The scattering curves and the envelopes computed from *Sa*Pdx1 in Tris–HCl buffer showed polydispersity and corresponded to a hexamer/dodecamer mixture, while the samples in sulfate and phosphate buffer were monodispersed and consistent with dodecamers in solution (Fig. 2 and Table S1). The *ab initio* models built from the scattering curves were compared with AlphaFold2 (AF2) models and revealed the expected quaternary structures with hexamers and dodecamers arranged in C6 and D6 symmetric assemblies (Fig. 2).

**Figure 3:**
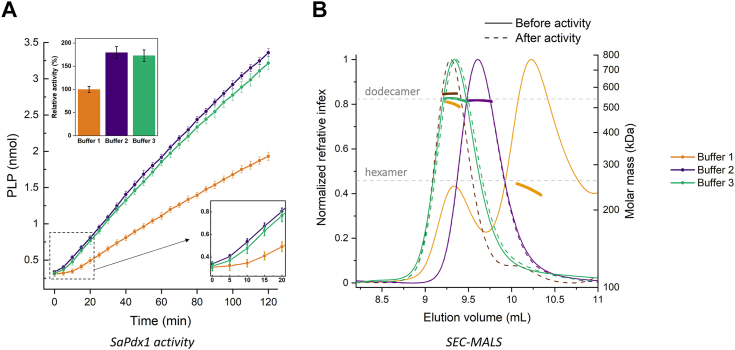
**PLP synthesis in different buffers.***A*, PLP synthesis by *Sa*Pdx1 in the presence of R5P, G3P, and ammonia. The activity measured in buffer 1 (50 mM Tris–HCl pH 8, 150 mM NaCl) was lower than that in buffer 2 (50 mM Tris–HCl pH 8, 200 mM Na_2_SO_4_) or buffer 3 (100 mM Na_2_HPO_4_ pH 8, 150 mM NaCl). The inset shows that the initial delay in enzyme activity is also increased when buffer 1 is used. These results were obtained from three independent experiments, each performed in triplicate. *B*, SEC-MALS analysis of *Sa*Pdx1 in different buffers. Prior to substrate addition, *Sa*Pdx1 was found to be dodecameric in buffers 2 and 3 and in equilibrium of hexamers and dodecamers in buffer 1, favoring hexamers. After adding the substrates R5P, G3P, and ammonia, the enzyme was found to be a dodecamer in all buffers evaluated. These results were obtained from two independent experiments. G3P, glyceraldehyde 3-phosphate; MALS, multiangle light scattering; PLP, pyridoxal 5-phosphate; R5P, ribose 5-phosphate; SEC, size-exclusion chromatography.

To further confirm the dodecameric species as the active species, the *Sa*Pdx1 enzyme purified in standard Tris–HCl buffer was incubated with its substrates, ammonia, R5P, and G3P, and the SEC/multiangle light scattering (MALS) profile was determined during the enzyme activity experiments. As shown in Figure 3, *Sa*Pdx1 is observed in the form of dodecamers in the presence of buffers 2 and 3 and in an equilibrium among hexamers and dodecamers, although favoring hexamers, when in the presence of buffer 1 (Tris–HCl). However, we clearly observed a shift in the profile, suggesting that after incubation with substrates, the dodecameric species was the preferential composition. Interestingly, neither R5P nor R5P + ammonia was sufficient to promote a complete shift for the dodecameric species, suggesting that, during catalysis, the maximal shift for the formation of the dodecameric state is achieved (Fig. S3).

In conclusion, we observed that *Sa*Pdx1 can form hexamers or dodecamers in solution, depending on the chemical environment. However, pyridoxal synthesis required the formation of the dodecamer species.

### Crystal structure of *Sa*Pdx1

Crystals of *Sa*Pdx1 were obtained, and a dataset was recorded at the MANACA beamline at the Brazilian Sirius light source. The structure was solved by molecular replacement with an AF2 model and refined to 3.0 Å resolution (Table S2). The asymmetric unit was composed of two *Sa*Pdx1 monomers, but the H32 crystal symmetry readily revealed the dodecameric structure of the PLP synthase enzyme.

The dodecamer forms a cylindrical quaternary structure with an opening diameter of approximately 52 Å and a lateral height of approximately 84 Å. Each monomer is folded as a slightly distorted eight-stranded TIM-barrel with the top of the barrel pointing toward the internal aperture of the cylindrical quaternary structure. The seventh strand in the barrel is sufficiently distorted to not be recognized as a beta strand in the visualization software Chimera (24, http://www.cgl.ucsf.edu/chimera). This distortion breaks the hydrogen bond pattern among strands 6 and 7, contributing to increased mobility in this region. As previously noted, this mobility allows the catalytic lysine K150 to move outward from the active site in the absence of the covalent intermediate I_320_ and may also facilitate the diffusion of ammonia to the active site (25). The experimental electron density allowed the modeling of residues 17 to 272 out of the 297 residues of the enzyme, with a missing region including seven residues in the ‹2′ region. This flexible region of the Plasmodial enzyme was shown to retain its flexibility in the absence of a substrate. However, it adopts a small helical structure when bound to Pdx2 and R5P (21).

**Figure 4:**
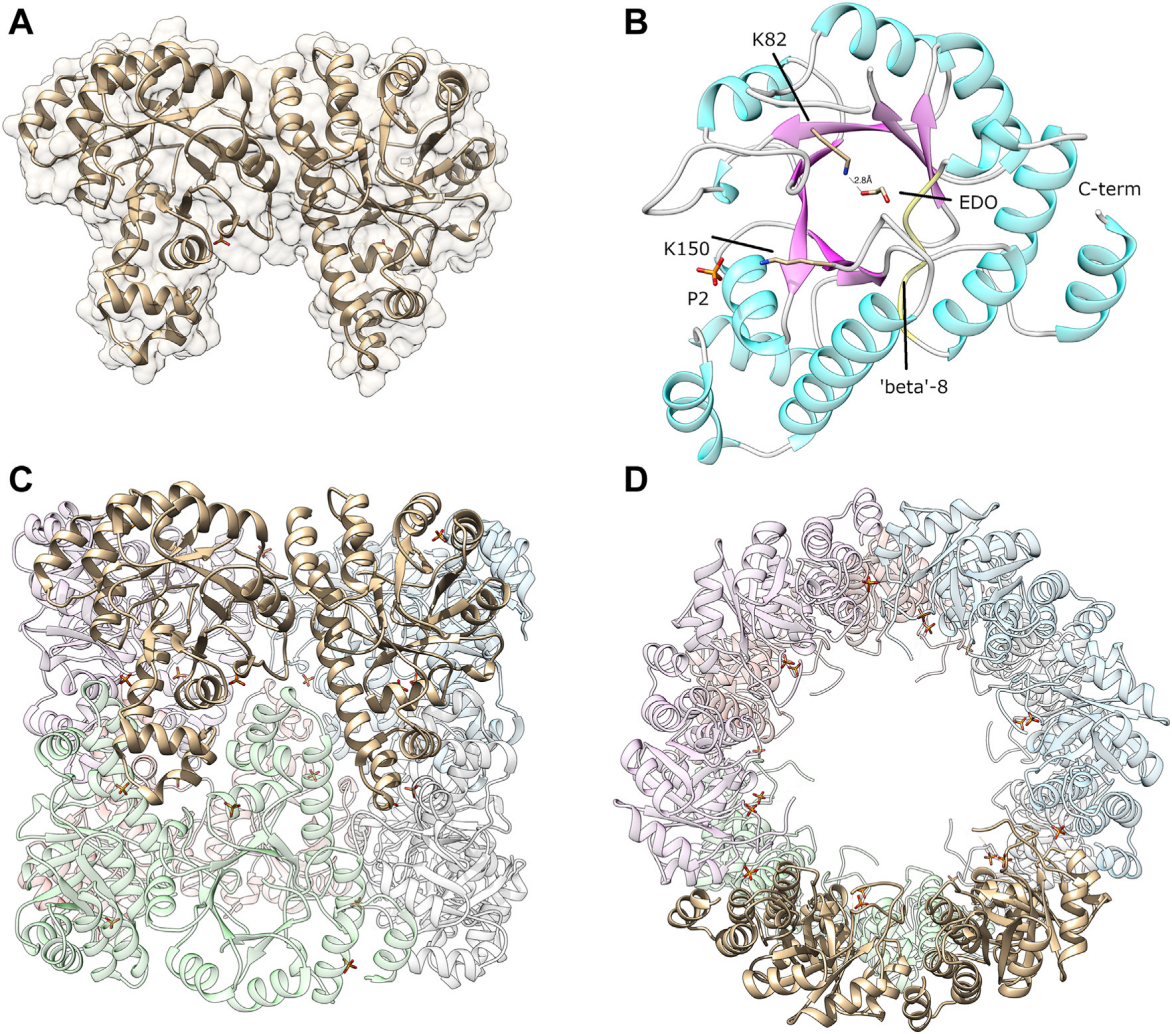
**Crystal structure of *Sa*Pdx1.***A*, the asymmetric unit content has two *Sa*Pdx1 monomers. *B*, a *closer view* of the *Sa*Pdx1 monomer: the distorted 8 strand is shown in *yellow*. An EDO molecule is shown in the active site interacting with lysine K82. The catalytic lysine K150 and the phosphate bound to the P2 site are indicated. *C* and *D*, two orthogonal views of the *Sa*Pdx1 dodecamer assembled by the H 32 crystal symmetry.

Two electron densities were identified in the P2 phosphate binding site, and the densities were modeled as phosphate molecules interacting with R138 and R139 from the LGEAx**RR**I sequence motif. Additionally, an ethylene glycol (EDO) molecule was observed to bind to the active site of *Sa*Pdx1 close to the catalytic lysine. For one of the monomers in the asymmetric unit (chain B), a hydrogen bond was clearly formed between K82 and the ethylene glycol molecule (Fig. 4*B*). Interestingly, independent crystal structures were obtained from crystals with different space groups. However, all the structures revealed the same quaternary arrangement and structural features.

### The *Sa*PLP complex is transiently formed

**Figure 5:**
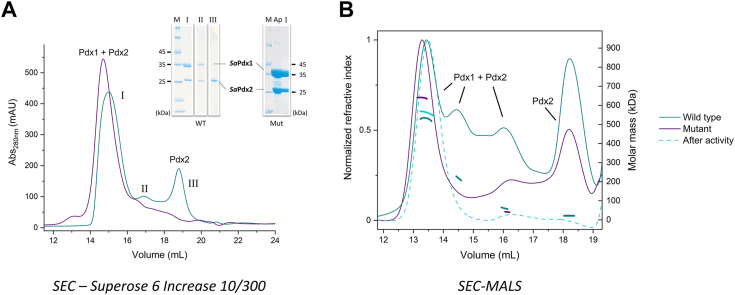
***Sa*PLP assembly.***A*, size-exclusion chromatography of the PLP complex in buffer 4 (50 mM Tris–HCl, pH 8, 150 mM NaCl, 10 mM L-glutamine). In *cyan lines*, the SEC profile for the *Sa*Pdx1-2_wt_. SDS‒PAGE (*inset*) shows that peaks I and II contain *Sa*Pdx1-2_wt_, while peak III contains free Pdx2_wt_. The *purple line* shows the SEC profile for *Sa*Pdx1-2_mut_ with a single peak containing the *Sa*Pdx1–*Sa*Pdx2_mut_ complex. The gel lane labeled as Ap shows the sample prior to SEC analysis, while the sample labeled as I shows *Sa*Pdx1–*Sa*Pdx2_mut_ complex. These results were obtained from five independent experiments. *B*, SEC-MALS analysis confirmed unsaturated *Sa*Pdx1-2_wt_ species, as well as some free *Sa*Pdx2 without mutation (*cyan line*). For *Sa*Pdx1-2_mut_ (*purple line*), the equilibrium is shifted toward the fully saturated PLP complex. After adding the substrates (*cyan dashed line*), *Sa*Pdx1-2_wt_ is also shifted toward a fully saturated complex. The results were obtained from two independent experiments. PLP, pyridoxal 5-phosphate; SAXS, small-angle X-ray scattering.

The interaction between Pdx1 and Pdx2 is reported to be transient, which makes crystallization experiments challenging (26). However, Strohmeier and coworkers observed that a mutation in one amino acid from the catalytic site of *Bs*Pdx2, H170N (H165N in *Sa*Pdx2), stabilizes the Pdx1/Pdx2 complex in a fully saturated form, enabling structural analysis (19). We used combinations of *Sa*Pdx1 and *Sa*Pdx2 in the absence (*Sa*Pdx1-2_wt_) and presence (*Sa*Pdx1-2_mut_) of the H165N mutation in *Sa*Pdx2. To confirm the *Sa*Pdx1/*Sa*Pdx2 assembly after mixing the independently purified enzymes, the SEC peaks were subjected to SDS‒PAGE analysis. As expected, the *Sa*Pdx1-2_mut_ assembly was fully saturated, as evidenced by the single peak eluted from SEC (Fig. 5*A*), unlike *Sa*Pdx1-2_wt_. The chromatogram (Fig. 5*A*) and SDS‒PAGE Lane II suggest that some Pdx2 molecules were attached to the native complex. SEC-MALS (Fig. 5*B*) also indicates that *Sa*Pdx1-2_wt_ can be observed in solution in undersaturated conditions.

**Figure 6:**
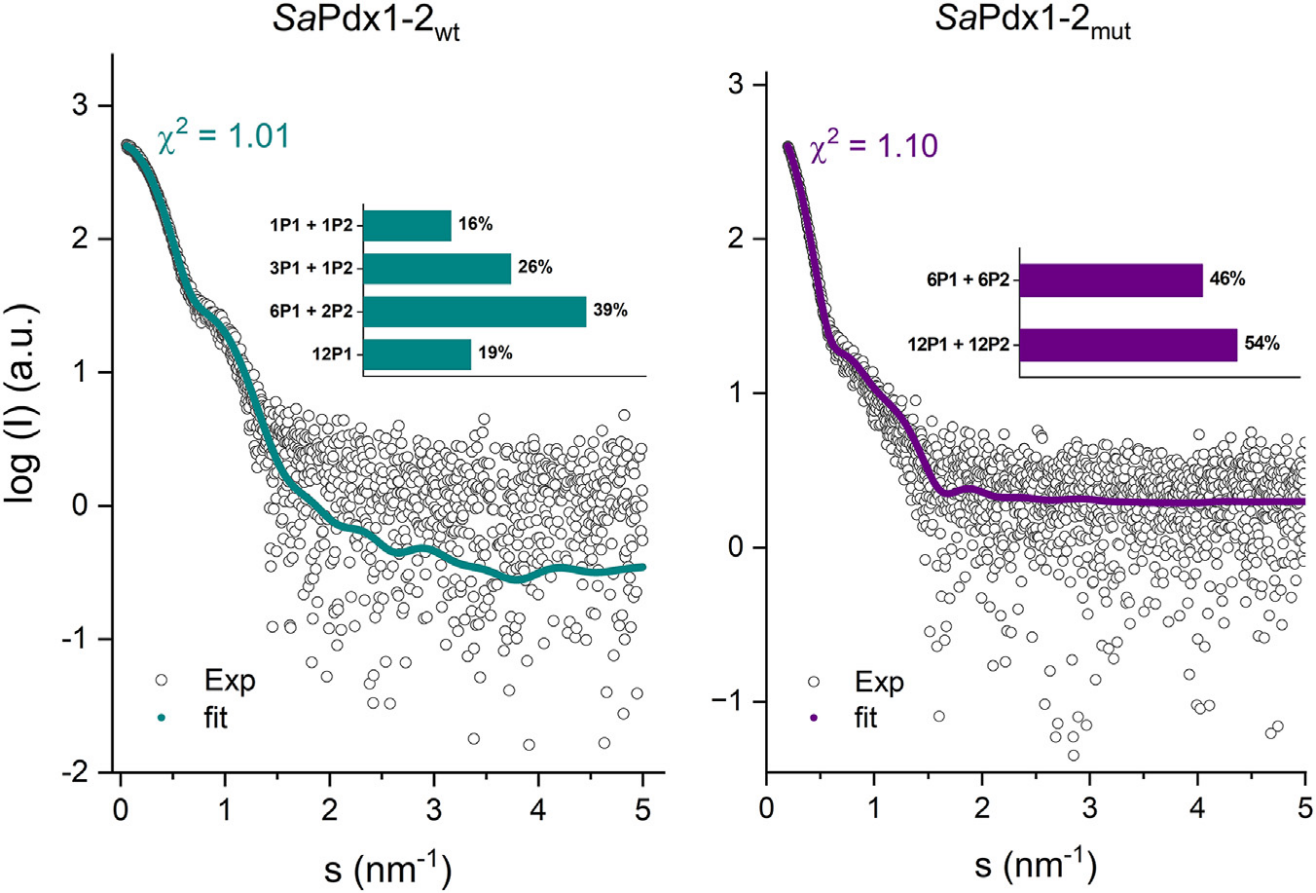
**SAXS analysis of the *Sa*PLP synthase complex obtained in SEC-SAXS measurements.***Left*: scattering data (*dots*) and best fit of the *Sa*Pdx1-2_wt_ sample composition (*cyan line*), as estimated by OLIGOMER. The composition is shown in the inset. *Right*: scattering data (*dots*) and best fit of the *Sa*Pdx1-2_mut_ composition (*purple line*), as estimated by OLIGOMER. The composition is shown in the inset. SaPLP, *Staphylococcus aureus* pyridoxal 5-phosphate; SAXS, small-angle X-ray scattering; SEC, size-exclusion chromatography.

The stoichiometry of the native PLP synthase was initially assessed by SAXS analysis. To the best of our knowledge, this is the first time a stoichiometric analysis has been performed for a native PLP synthase complex. First, the experimental scattering curves were compared with the calculated scattering amplitudes of the built models of the *Sa*Pdx1–*Sa*Pdx2 complex, with different stoichiometries, using the AF2 3D models of *Sa*Pdx1 and *Sa*Pdx2 superimposed with the crystallographic 24-mer structure of *B. subtilis* PLP synthase (PDB: 2NV2). The software OLIGOMER (27) was used to estimate the stoichiometry of each WT and mutant complex; the data are shown in Table 2, and the best fits are shown in Figure 6.

**Table 2:** SaPLP synthase WT and mutant SAXS data

Parameter	WT	Mutant
Data collection		
Beamline	P12 (PETRA III/DESY)	
Wavelength (Å)	1.2398	
q range (Å^−1^)	0.004–0.74	
Structural parameters		
Guinier R_g_ (nm)	4.50 ± 0.02	5.76 ± 0.01
sRg limits	0.28–1.29	0.30–1.30
P(r) R_g_ (nm)	4.50	5.74
D_max_ (nm)	13.2	18.2
Porod volume (Å³)	250,298	827,981
OLIGOMER *χ*^2^ fit	1.01	1.10
Molecular weight determination^a^		
Heterodimer theoretical MW (kDa)	55	
Porod volume (kDa)	160	517
Bayesian (kDa)	186	480

SaPLP, *Staphylococcus aureus* pyridoxal 5-phosphate; SAXS, small-angle X-ray scattering.

a The molecular weight was calculated using two different methods: the Porod Volume divided by 1.6 and a Bayesian inference. Both are concentration-independent.

Interestingly, the SAXS data confirm that the interaction of *Sa*Pdx1 and WT *Sa*Pdx2 is transient. For the WT complex, most of the sample consists of substoichiometric associations of *Sa*Pdx1 and *Sa*Pdx2_wt_ in a 6:2 or 3:1 ratio. *Sa*Pdx1 alone in dodecameric form was also observed in almost 20% of the sample.

On the other hand, in the mutant complex, a saturated (1:1) association of *Sa*Pdx1 and *Sa*Pdx2_mut_ is observed for the sample, with 46% and 54% of the complexes in the hexameric and dodecameric forms, respectively (Fig. 5).

SEC-MALS measurements were also used to assess the stoichiometry of the *Sa*Pdx1-*Sa*Pdx2 association. As previously observed in the right panel of Figure 5, the WT complex exhibited multiple elution peaks with corresponding molar masses of 530 kDa (41%), 220 kDa (11.5%), 61 kDa (12.5%), and 21 kDa (35%). The last peak corresponds to isolated *Sa*Pdx2, as seen in SEC peak III (Fig. 5). Despite the lack of a higher resolution among the peaks, these results were consistent with the findings from SAXS analysis. In the case of the mutant complex, a fully saturated complex (610 kDa) was observed. Additionally, there was evidence of a partial assembly or a dimer of *Sa*Pdx2_mut_ (46 kDa) and a third peak corresponding to the monomer (22 kDa), as it was intentionally added in excess for the measurement.

The most significant and previously unreported finding was the behavior of the WT complex after catalytic activity. It was evident that there was a shift to a single peak corresponding to the *Sa*Pdx1 dodecameric core fully saturated with *Sa*Pdx2. This empirical evidence supports the hypothesis that the WT complex becomes fully saturated only during catalysis or artificially by Pdx2 mutation.

These observations made in solution suggest that the association between Pdx1 and Pdx2 may be transitory and that the 12:12 stoichiometry (Pdx1:Pdx2) is achieved only for catalysis or artificially by Pdx2 mutation.

### Crystal structure of the *Sa*PLP complex

The initial crystals obtained for *Sa*Pdx1-2_wt_ and *Sa*Pdx1-2_mut_ were not suitable for X-ray diffraction. To optimize the crystallization, seeding experiments were performed. After monitoring the early stage of crystallization and despite all efforts, the diffraction data could only be collected up to 4.0 Å resolution for the *Sa*Pdx1-2_wt_ complex and 3.0 Å resolution for the mutant complex (Table S2).

After data processing for the WT complex, the molecular replacement search function in Phaser (28) as implemented in Phenix (29) readily identified 12 solutions for *Sa*Pdx1 with a final translation function Z-score of 22.4 and a log-likelihood gain of 2508. However, the composition in the asymmetric unit with *12 Sa*Pdx1 and *12 Sa*Pdx2 would result in only 28% solvent in the unit cell, which is unrealistic. In fact, Phaser could not locate any *Sa*Pdx2, suggesting that *Sa*Pdx2 was not bound or at least was not bound with sufficient occupancy to be identified in the molecular replacement search.

**Figure 7:**
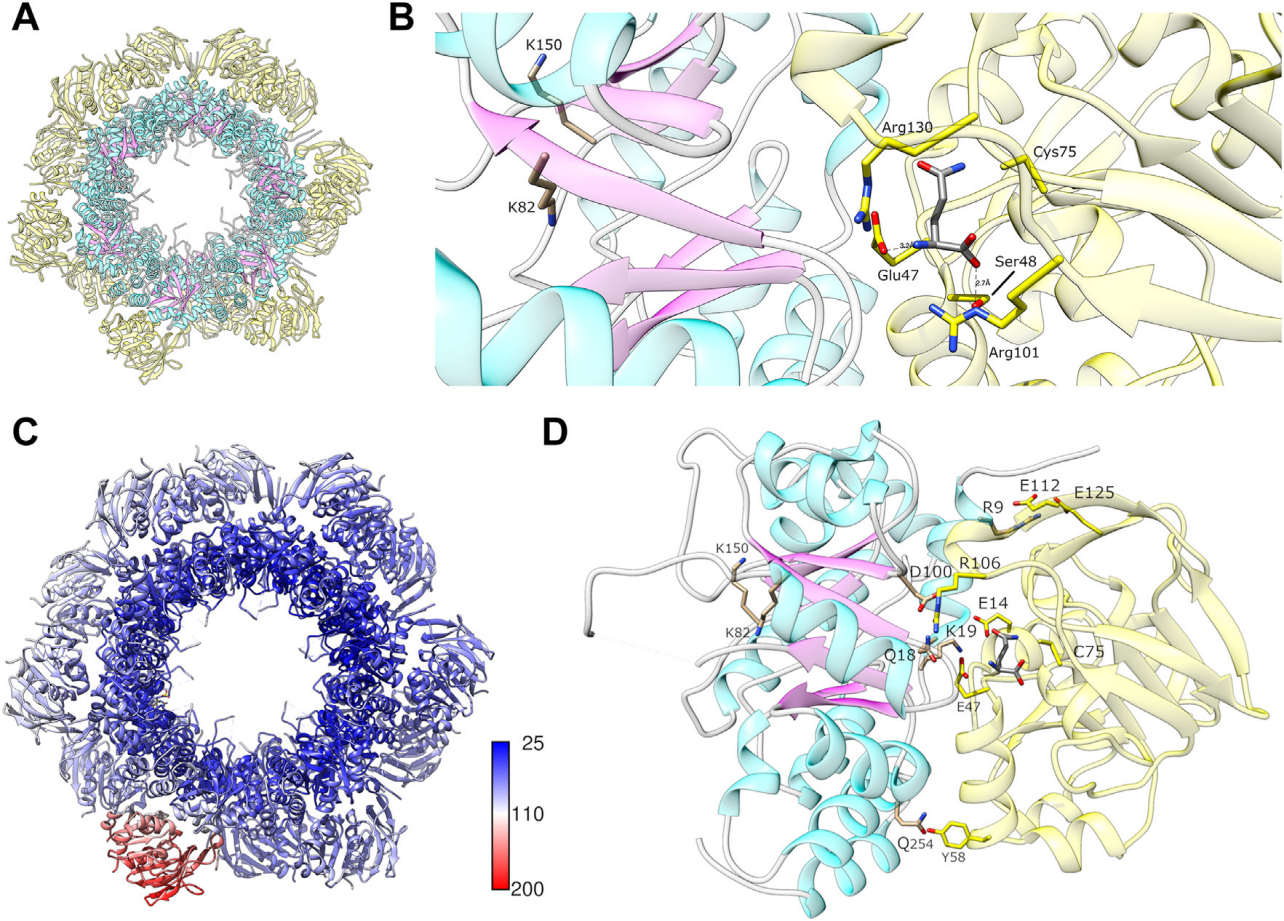
**Crystal structure of the *Sa*PLP complex.***A*, overall arrangement of the complex. *Sa*Pdx1 is shown *colored* by secondary structure, while *Sa*Pdx2_mut_ is shown as a *yellow cartoon*. *B*, a glutamine amino acid (*gray sticks*) is bound to the *Sa*Pdx2_mut_ active site, shown in *yellow*. *C*, crystal structure of the *Sa*PLP complex colored by crystallographic B factors. A color key is shown in Å^2^ units. *D*, a detailed view of the interactions between *Sa*Pdx1 and *Sa*Pdx2_mut_ in the *Sa*PLP complex. SaPLP, *Staphylococcus aureus* pyridoxal 5-phosphate.

The diffraction data of the mutant complex were processed up to 3.0 Å resolution. The phaser found a solution with a final translation function Z-score/a log-likelihood gain of 18.5/1565, containing 12 *Sa*Pdx1 and 11 *Sa*Pdx2_mut_. However, during the refinement, an electron density for the 12th *Sa*Pdx2_mut_ chain allowed the tracing of the chain, although with missing parts. The model was refined in Phenix.refine (30), and the final model was validated with MolProbity (31). The final model for the *Sa*PLP_mut_ complex showed remarkable structural conservation of the *Sa*Pdx1 dodecamer with minimal structural rearrangements after *Sa*Pdx2_mut_ binding, as suggests the comparison of Figures 4*D* and 7*A*. The alignment of the two structures revealed an RMSD of only 0.5 Å for over 17,700 atoms. As compared to the AF2 model, as used to fit in the SAXS envelope, the experimental structure shows a remarkable structural similarity. A complex composed of a single chain of *Sa*Pdx1 and a single chain of *Sa*Pdx2 predicted by AF2 showed an RMSD of 0.63 Å. The same comparison with the plasmodial PLP synthase complex (PDB 4ADS (21)) reveals an RMSD of 1.13 Å. A comparison with the *Arabidopsis thaliana* Pdx1 core (PDB 5LNS (25)) reveals a similar conservation with an RMD of 1.10 Å for a Pdx1 dimer along the 2-fold axis of the D6 symmetric arrangement. A similar conservation is observed with the *S. cerevisiae* Pdx1 structure (PDB 3O06 (32)) with a trimer within the hexameric arrangement showing an RMSD of 0.59 Å.

*Sa*Pdx2_mut_ interacts with *Sa*Pdx1 through a network of polar interactions, including *Sa*Pdx1 Q254 and *Sa*Pdx2 Y58 (2.6 Å), K19 and E14 (3.3 Å), D100 and R106 (2.8 Å), Q18 and E47 (3.1 Å), R9 and E112 (2.7 Å), and R9 and E125 (2.8 Å), as shown in Figure 7*D*. According to PDBePISA (33), the 12 interfaces among *Sa*Pdx1 and *Sa*Pdx2_mut_ have an average interface area of approx. 1424 Å^2^, with 23 hydrogen bonds and ten salt bridges, supporting the formation of the Pdx1–Pdx2 complex.

A glutamine residue was observed in the active site of *Sa*Pdx2_mut_. The Nε atom from glutamine interacts with the catalytic cysteine C75 (2.3 Å), as shown in Figure 7*B*. Other interactions include S48 interacting with the carboxylate group of glutamine (2.7 Å) and E47 interacting with the amine group (3.2 Å). The glutamine bound to SaPdx2_mut_ is approximately 23 Å apart from *Sa*Pdx1 K82 (measured from the NZ atom). This distance is measured through the interior of the barrel of *Sa*Pdx1 in a pathway usually described as the *ammonia tunnel*, where the ammonia molecule is thought to diffuse until reaching the *Sa*Pdx1 active site. As previously mentioned, diffusion through this path must be more efficient than diffusion from the environment since the *Sa*PLP complex is more active than *Sa*Pdx1 + ammonia, as shown in Figure 1.

Unexpectedly, for one of the 12 *Sa*Pdx2_mut_ molecules bound to *Sa*Pdx1, the electron density was observed to be rather weak. This weak density resulted in higher B factors for this particular *Sa*Pdx2_mut_ chain than the other *Sa*Pdx2_mut_ chains. The *Sa*PLP_mut_ complex colored by the B factor is shown in Figure 7*C* and reveals the increased temperature factors for this *Sa*Pdx2_mut_ chain. This weak density can be understood as a low occupancy chain in the complex among the asymmetric units, again reinforcing that, even in the presence of the mutation H165N in *Sa*Pdx2, the complex may be formed under the stoichiometric association of *Sa*Pdx1 and *Sa*Pdx2_mut_.

## Discussion

The PLP synthase complex is an interesting and intriguing molecular machine. Despite its relevance for organisms from archaea to metazoans and its potential role as a target for antibiotic discovery, many aspects related to the activation of the synthase complex remain unknown. Rodrigues and coworkers completed a *tour de force* of the structural characterization of snapshots of the synthesis reaction for *Arabidopsis* Pdx1 (25, 34). Their work elucidated and highlighted a number of distinct aspects of the enzyme mechanism and provided a structural basis for the synthesis of PLP. Many questions, however, remain open, including aspects related to the dynamics of complex assembly and PLP synthesis.

The relevance of Pdx2 in the *Sa*PLP complex is evident from the biochemical data, which show that *Sa*Pdx2 in complex with *Sa*Pdx1 enhances the efficiency for PLP synthesis up to 22 times when compared to *Sa*Pdx1 alone or in the presence of ammonia. The previously identified ammonia channel, a cavity of approx. 23 Å in the Pdx1–Pdx2 complex connecting the active sites for these enzymes, explains this difference by providing a rapid delivery of ammonia to the synthesis reaction. Delivery through the ammonia channel is expected to be more efficient than diffusion, thus providing selective pressure for maintaining the association of Pdx1 and Pdx2 during evolution. Curiously, when the G3P concentration is kept constant and the R5P concentration is varied, the increase in efficiency due to *Sa*Pdx2 is mostly due to the decrease in K_M_, while when the G3P concentration is varied, the increase in efficiency is mostly due to *k*_*cat*_. These findings are in line with the recent results by Rodrigues and coworkers (34), who showed that ammonia is required for the reaction to proceed after R5P covalently bound to lysine K82 (*Sa*Pdx1 numbering) and prior to the formation of the intermediate I_320_. In this context, the more efficient delivery of ammonia may result in a more efficient enzyme reaction progress and more efficient binding of more substrate (R5P).

The effect of ammonia or Pdx2 on the synthesis of PLP is rather diverse. Müller and coworkers observed that the synthase activity of *Plasmodium falciparum* Pdx1 was the same in the presence of Pdx2 or in the presence of ammonia (35). On the other hand, for the *Mycobacterium tuberculosis* Pdx1, replacing Pdx2 with ammonia results in 3-fold lower activity (36). These measurements were made to determine the specific activity. Here, using the same parameter to assess the enzyme activity, we found that replacing *Sa*Pdx2 with ammonia resulted in 2.5-fold lower activity in PLP synthesis. By measuring the Michaelis–Menten kinetic parameters, we observed a 5-fold or 20-fold reduction in activity as a function of G3P or R5P concentration, respectively. These findings suggest that orthologous Pdx1 enzymes may have different sensibility to ammonia or Pdx2 for the *de novo* synthesis of PLP.

As previously observed for bacterial PLP complexes (19), we found that *Sa*Pdx1 can form two major species in solution, identified in SEC, SEC-MALS and SAXS data as hexameric and dodecameric species. We also observed changes in the composition of the species in solution by changing the buffer, indicating that changes in the environment can affect the assembly of *Sa*Pdx1. On the other hand, the active enzyme in the presence of R5P, G3P, and ammonia shifts its equilibrium toward the dodecameric species, confirming it as the active arrangement. These findings align with previous observations in *B. subtilis* (19) and Plasmodial Pdx1 (21). However, our study expands the understanding of assembly regulation by demonstrating the influence of the chemical environment. In conclusion, while Pdx1 for different organisms can exist as hexamers, dodecamers, or equilibrium of both, the presence of substrates consistently appears to drive the formation of the dodecameric species, which likely represents the active form.

We could not detect any allosteric regulation among the Pdx1 monomers, suggesting that the reaction might be catalyzed in an independent manner by each monomer in the dodecamer. Why does the enzyme form a higher oligomeric species? We believe that the requirement of a higher-order oligomeric species is more related to the efficient delivery of ammonia by Pdx2 than to PLP synthesis itself. However, more investigations on this topic are still necessary.

Our crystal structure of the *Sa*PLP_mut_ complex showed a lower occupancy for one out of the 12 *Sa*Pdx2_mut_ chains. This finding, although unexpected, is consistent with the transient association of Pdx2 and Pdx1. As previously suggested, Pdx2 activation requires interaction with Pdx1 and the latter requires ammonia to be delivered by Pdx2. The data provided here by crystal structures and solution scattering together with biochemical data allow us to propose a model in which Pdx2 is constantly interacting with Pdx1, being activated, delivering ammonia, and then dissociating in a highly dynamical complex formation. In this context, taking an average over time, the ratio between Pdx2 and Pdx1 in the complex will always be substoichiometric, as we observed in the SAXS data. The mutation in Pdx2 stabilizes the complex and has been used for the structure determination of other PLP complexes (21). *S. aureus* Pdx2_mut_ increased the stability of *Sa*PLP compared to the WT complex. The relatively low-resolution diffraction observed for *Sa*PLP_wt_ crystals together with the analysis of the unit cell content suggest that one or two Pdx2 molecules might be bound to the Pdx1 dodecamer. However, the low occupancy and the probable mixing of binding sites impair the determination of the complex structure. These experimental pieces of evidence are in agreement with the highly dynamic assembly of the *Sa*PLP complex.

In terms of drug discovery investigations, the model proposed here is also of interest. The dynamic assembly of the PLP complex may suggest that the interaction between Pdx1 and Pdx2 should be weak enough to allow rapid association and dissociation. This weak yet necessary interaction presents an intriguing opportunity for the design of inhibitors that can disrupt the association between Pdx2 and Pdx1, consequently inhibiting the synthesis of vitamin B6. Although this potential has not been explored extensively, it opens new avenues for the development of therapeutic agents that could target this interaction and potentially modulate the production of vitamin B6. Additionally, the gained structural insights also open the fascinating option to design modified substrates that can be employed in suicide drug discovery to poison PLP-dependent enzymes. Further investigation and research in this area could lead to the discovery of novel inhibitors and contribute to the development of new antibiotic strategies.

## Experimental procedures

### Molecular biology

The coding sequences of the Pdx1 and Pdx2 enzymes from *S. aureus* were synthesized by Biomatik and cloned separately into pETTRXA-1a/LIC and pETM11/LIC expression vectors. Cloning was performed by the ligation-independent cloning LIC method, as described by Camilo & Polikarpov (37). Cloning into these vectors allows expression of the protein(s) with an N-terminal 6xHis tag and tobacco etch virus (TEV) protease cleavage site fusion (6xHis-TEV) in the pETM11/LIC vector and an N-terminal 6xHis tag with thioredoxin (TRX) and TEV site fusion (6xHis-TRX-TEV) in the pETTRXA-1a/LIC vector. Site-directed mutagenesis of *Sa*Pdx2_H165N_ was carried out by PCR using the construct pETM11::*Sa*Pdx2 as a template. The following oligonucleotides were used: 5′ primer GGTGTGAGTTTTAATCCGGAAC and 3′ primer CAGATATTTGCCCTGCTGC.

### Protein expression and purification

Chemically competent *E. coli* Rosetta (DE3) cells were transformed with the constructs and grown in LB medium containing antibiotics at 37 °C to an absorbance (*A*) of 0.6. Protein expression was induced with 0.5 mM IPTG and then grown for 16 h at 18 °C. The 1 L cell pellet was resuspended in 40 ml of ice-cold purification buffer (50 mM Tris–HCl buffer, pH 8, 300 mM NaCl) containing 5 mM β-mercaptoethanol, 1 mM PMSF, and 10% glycerol. Lysozyme (0.3 mg/ml) was added to the cell suspension, which was lysed by ultrasonication. The cell debris was removed by centrifugation (20,000*g* for 30 min at 4 °C), and the clarified lysate was applied to a gravity flow column containing 5 ml nickel-charged nitrilotriacetic acid agarose resin (QIAGEN) preequilibrated with purification buffer. The column was washed twice with purification buffer containing 10 mM and 25 mM imidazole. The bound proteins were eluted with purification buffer containing 250 mM imidazole and dialyzed using elution buffer 1 (50 mM Tris–HCl, pH 8, 150 mM NaCl, 1 mM EDTA, 2 mM DTT) or buffer 2 (50 mM Tris–HCl, pH 8, 200 mM Na_2_SO_4_, 1 mM EDTA, 2 mM DTT) or buffer 3 (100 mM Na_2_HPO_4_ pH 8, 150 mM NaCl, 1 mM EDTA, 2 mM DTT). Afterward, the fusion protein was cleaved with TEV protease (1 mg TEV:10 mg protein) for 16 h at 4 °C. A second nickel affinity chromatography step was performed with digestion, and the eluate was concentrated and subsequently submitted to SEC. For *Sa*Pdx1 purification, a HiLoad 16/600 Superdex 200 pg column was used, and a Superdex 75 pg column was used for *Sa*Pdx2 (both GE Healthcare). For complex formation, highly pure *Sa*Pdx1 and *Sa*Pdx2 (WT or mutant) were mixed in a 1:1 M ratio in buffer 1 containing 10 mM L-glutamine (buffer 4) and incubated for 16 h at 4 °C. The *Sa*Pdx1/*Sa*Pdx2 assembly was purified by gel filtration on a Superose 6 Increase 10/300 Gl column (GE Healthcare) with buffer 4. The purified proteins were analyzed by 15% SDS‒PAGE and Coomassie blue staining.

### Biochemical analyses

The activity of PLP synthase was measured using a Tecan Infinite F200 PRO microplate spectrophotometer (Thermo Fisher Scientific) with a 96-well plate (Greiner Bio-One). Reactions were performed at 37 °C in 50 mM Tris–HCl pH 8 and 150 mM NaCl. The reaction mixture included 40 μM free Pdx1 and/or 40 μM Pdx2, 1 mM R5P and 1 mM G3P. Additionally, 20 mM glutamine or 20 mM (NH_4_)_2_SO_4_ was added to the *Sa*PLP complex or isolated *Sa*Pdx1, respectively. The product PLP formation was monitored at 414 nm, the wavelength where a Schiff’s base, formed by PLP and the primary amine of the Tris buffer, was observed.

### SEC coupled with MALS

For SEC-MALS measurements, 50 μl of each sample (∼6 mg/ml) was loaded into Superdex 200 10/300 (for Pdx1 samples) or Superose 6 Increase 10/300 (for complex measurements) columns (GE Healthcare) by HPLC on a Waters 600 Controller, following the protocol of the manufacturer and using a flow rate of 0.5 ml/min at 20 °C. After SEC, the sample was loaded into an in-line DAWN TREOS miniature system equipped with a refractive index OptiLab T-REX detector (Wyatt Technology). Data analysis was performed using ASTRA 7 software (Wyatt Technology, http://www.wyatt.com/products/software/astra.html).

### Small-angle X-ray scattering

To prepare samples for batch SAXS measurements, the protein samples were purified by SEC using the buffer of choice. The dialysis buffer was kept for the SAXS buffer subtraction measurements. Two different concentrations of *Sa*Pdx1 that is 3 and 6 mg/ml were measured. The data were collected at the EMBL beamline P12 (PETRA III) with an automated robotic sample changer and a Dectris 2D photon-counting detector (PILATUS-6 M) with a 3.0 m sample-to-detector distance and X-rays with a wavelength of 1.2398 Å (photon energy 10 keV). SEC combined with SAXS experiments were performed with a protein concentration of 7 mg/ml. Samples were injected into a pre-equilibrated Superdex 200 Increase 10/300 column (GE Healthcare) for Pdx1 and Superose 6 Increase 10/300 (GE Healthcare) for the PLP complex with a flow rate of 0.5 ml/min. Data processing was carried out using ATSAS 3.1.3 software (EMBL) (38). To minimize background noise, buffer frames were manually selected and subtracted from each elution peak.

Guinier analysis was performed on the averaged and normalized curves to determine the *R*_*g*_ and the intensity at zero angle (*I(0)*), for data points within the range *qR*_*g*_ < 1.3. Distance distribution function was carried out to obtain the maximum particle dimension (*D*_*max*_). The generated GNOM file was subsequently employed for *ab initio* modeling. Low-resolution *ab initio* models were generated using the DAMMIN (39) and GASBOR (40) programs. To generate the GASBOR models, the symmetry imposed was P6 for hexameric models and P62 for dodecameric models.

Additionally, the program OLIGOMER (41) was employed to estimate the volume fraction of different stoichiometries within the *Sa*PLP synthase WT and mutant complexes. To determine the stoichiometries tested in OLIGOMER theoretical scattering curves of atomic models obtained from AF2 modeling were calculated, compared, and fitted to the experimental data using CRYSOL (42) and SREFLEX (43).

### Crystallization and data collection

Crystallization screens for *Sa*Pdx1 were set using a protein concentration of 15 mg/ml and the commercial screening kits PACT Premier HTS and Morpheus III (Molecular Dimensions). After 5 days, numerous crystals appeared under different conditions. Crystals obtained from the Morpheus III-A11 condition (0.1 M Tris base; BICINE, pH 8.5; 1.6% w/v dipeptides mix; 20% v/v glycerol; 10% w/v PEG 4000) were flash-frozen in liquid nitrogen for data collection at the MANACA beamline facility of the Sirius Brazilian Synchrotron (LNLS).

For *Sa*Pdx1-2_wt_ and *Sa*Pdx1-2_mut_ complexes, initial crystallization conditions were established by applying commercially available crystallization screens (Molecular Dimensions) utilizing Zinsser Pipetting robots Honeybee 961 (Zinsser Analytic GmbH). The best conditions selected for optimization were PACT Premier HTS-H9 for *Sa*Pdx1-2_wt_ and a literature-described condition for *Sa*Pdx1-2_mut_ (5% PEG4000, 0.2 M triammonium citrate pH 7, 10 mM L-glutamine). Before data collection, crystals were briefly soaked in cryoprotected shock-frozen liquid nitrogen. The X-ray diffraction data from the *Sa*Pdx1–*Sa*Pdx2 complexes were collected at beamlines P11 (PETRA III/DESY) (44, 45) and EMBL P13 (PETRA III/DESY) (46).

The collected datasets were processed using the autoPROC package (47) with the following high-resolution cut-off criteria: R_pim_ ≤ 0.6; I/σ(I) ≥ 2; CC_1/2_ ≥ 0.3 for classical isotropic treatment or local (I/σ(I)) ≥ 1.2 for anisotropic analysis. The phase problem was solved by molecular replacement using Phenix suite (29). Model refinement was performed using phenix.refine (30) and COOT (48). *Sa*Pdx1 and *Sa*Pdx1-2_mut_ structures were deposited in the PDB with PDB Ids 8U9E and 8U7J, respectively.

## Data availability

All data are available in the main text or the supplementary materials. Crystal structures and their respective structure factors are deposited in the PDB, with PDB IDs 8U9E and 8U7J for *Sa*Pdx1 and *Sa*Pdx1-2_mut_ structure, respectively.

## Supporting information

This article contains supporting information (49, 50).

## Conflict of interest

The authors declare that they have no conflicts of interest with the contents of this article.
