# Supplementary material for: Structure and dynamics of the staphylococcal pyridoxal 5-phosphate synthase complex reveal transient interactions at the enzyme interface
Source: J Biol Chem. 2024 May 21;300(6):107404. doi: 10.1016/j.jbc.2024.107404 (PMC11237949; doi:10.1016/j.jbc.2024.107404)

**Structure and dynamics of the staphylococcal pyridoxal 5-phosphate synthase complex reveal transient interactions at the enzyme interface**

**Angélica Luana C. Barra^1,2^, Najeeb Ullah^2,3^, Hévila Brognaro^2^, Raissa F. Gutierrez^1^, Carsten Wrenger^4*^, Christian Betzel^2,4*^, Alessandro S. Nascimento^1*^**

1. São Carlos Institute of Physics, University of São Paulo, São Carlos, Brazil.

2. Institute of Biochemistry and Molecular Biology, Laboratory for Structural Biology of Infection and Inflammation, University of Hamburg, Hamburg, Germany.

3. Department of Biochemistry, Bahauddin Zakariya University, Multan, Pakistan

4. Unit for Drug Discovery, Department of Parasitology, Institute of Biomedical Sciences, University of São Paulo, São Paulo, Brazil.

* Correspondence: [cwrenger@icb.usp.br](mailto:cwrenger@icb.usp.br), [christian.betzel@uni-hamburg.de](mailto:christian.betzel@uni-hamburg.de) and [asnascimento@ifsc.usp.br](mailto:asnascimento@ifsc.usp.br)

**Supplementary Figure S1**. (a) Nickel affinity chromatography (1) supernatant of E. coli lysate; (2) flowthrough; (3)-(4) first and second wash with purification buffer without and with 25 mM of imidazole; (5)-(8) eluates with 100, 200, 300 and 500 mM of imidazole. (b) TEV protease cleavage (9) purified His6-TRX-SaPdx1 protein; (10) TEV protease cleavage solution; (11) SaPdx1 at the flowthrough. (M) protein molecular weight marker. 12% SDS-PAGE gel. (c) SEC in Buffer 1, chromatogram of free-tag SaPdx1 purification by HiLoad 16/600 Superdex 200 pg column; inset (12)-(14) SaPdx1 peak elution from 68-70 mL. 12% SDS-PAGE gel.


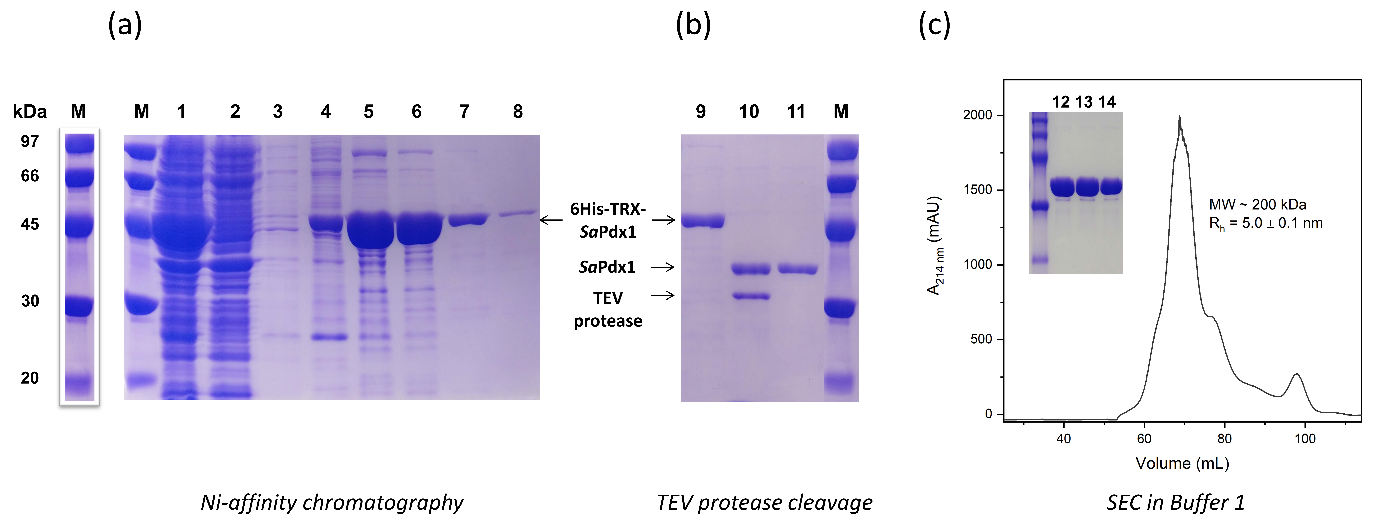


**Supplementary Figure S2**. Investigation of SaPdx1 oligomerization in the presence of sulfate and phosphate ions. Size distribution of SaPdx1 (X-axis) in (a) buffer 1 (50 mM Tris-HCl pH 8, 150 mM NaCl), (b) buffer 2 (50 mM Tris-HCl pH 8, 200 mM Na2SO4) and (c) buffer 3 (100 mM Na2HPO4 pH 8, 150 mM NaCl) over time (s) (Y-axis). The color intensity from blue to red demonstrates the increase in abundance. The calculated values of Rh and MW are given.


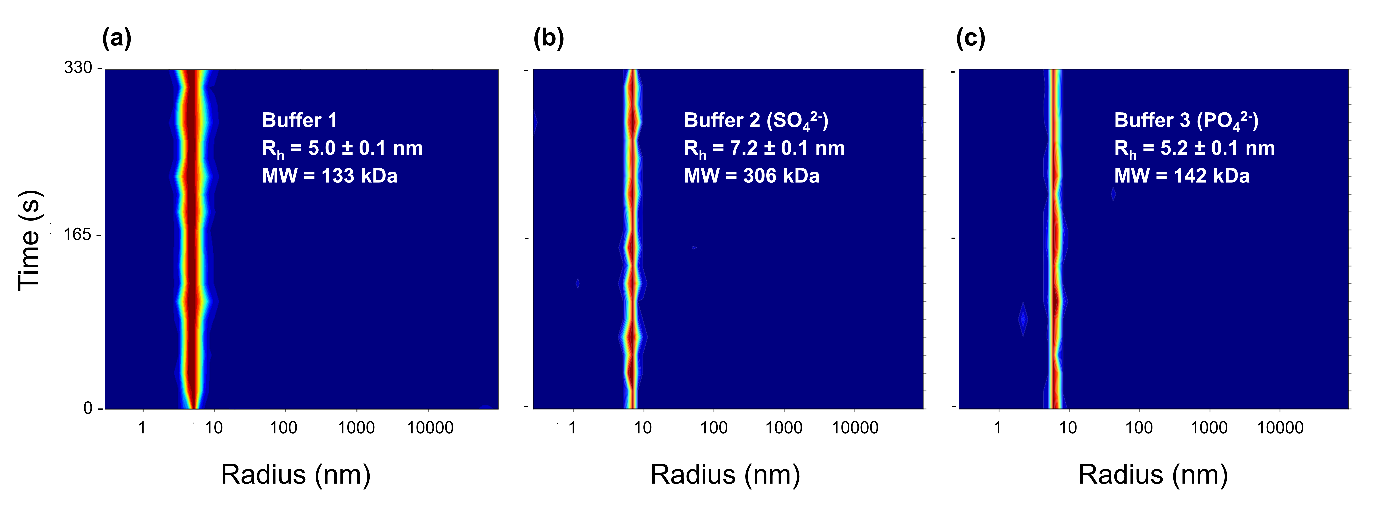


**Supplementary Table S**1. SaPdx1 SAXS data and structural parameters.

|  | **Buffer 1** | **Buffer 2** | | **Buffer 3** | |
| --- | --- | --- | --- | --- | --- |
| **Data collection** | | | | | |
| Beamline | P12 (PETRA III/DESY) | | | | |
| Wavelength (Å) | 1.2398 | | | | |
| q Range (Å^-1^) | 0.004 – 0.74 | | | | |
| Collection mode | Batch | | SEC-SAXS | |  |
| **Structural parameters** | | | | | |
| Guinier R_g_ (nm) | 4.53 ± 0.02 | 5.13 ± 0.02 | | 4.97 ± 0.01 | |
| sRg limits | 0.33 - 1.29 | 0.52 - 1.29 | | 0.30 - 1.30 | |
| P(r) R_g_ (nm) | 4.95 | 4.97 | | 4.88 | |
| D_max_ (nm) | 18 | 15 | | 14 | |
| Porod volume (Å³) | 259694 | 527981 | | 607399 | |
| GASBOR model *χ*² fit | 1.1 | 1.3 | | 1.1 | |
| **Molecular Weight determination *^a^*** | | | | | |
| Monomer theoretical MW (kDa) | 32 | | | | |
| Porod volume (kDa) | 162 | 330 | | 380 | |
| Bayesian (kDa) | 208 | 319 | | 319 | |

*^a^* The molecular weight was calculated using two different methods. The first uses the Porod Volume divide by 1.6 (44) and the second the Bayesian Interference (45). Both are concentration independent.

**Supplementary Table S2**. Data collection and refinement statistics for SaPdx1 and SaPdx1-2mut.

|  | **SaPdx1-2_mut_** | **SaPdx1** | **SaPdx1 DESY** |
| --- | --- | --- | --- |
| **Wavelength (Å)** | 1.033 | 0.977 | 1.0 |
| **Resolution range (Å)** | 54.83 - 3.019 (3.389 - 3.019) | 82.234 - 2.959 (3.01 - 2.959) | 49.8-2.827 (2.928-2.827) |
| **Space group** | P 1 | R 3 2 :H | R 3 2 : H |
| **Unit cell (Å,°)** | 101.235 132.374 142.097 106.49 109.51 105.56 | 182.407 182.407 96.626  90.0 90.0 120.0 | 192.419 192.419 448.198  90 90 120 |
| **Total reflections** | 272713 (13697) | 386679 (21276) | 782916 (73691) |
| **Unique reflections** | 71124 (3556) | 12897 (640) | 76173 (7482) |
| **Multiplicity** | 3.8 (3.9) | 30.0 (33.2) | 10.3 (9.9) |
| **Completeness (%)** | 59.3 (10.1) | 100.0 (100.00) | 99.59 (98.36) |
| **Ellipsoidal Completeness (%)** | 85.8 (64.2) | - | - |
| **Mean I/sigma(I)** | 4.7 (1.6) | 17.1 (2.2) | 16.04 (3.54) |
| **Wilson B-factor** | 69.51 | 92.08 | 56.98 |
| **R_merge_** | 0.127 (0.561) | 0.153 (2.058) | 0.1081 (0.5976) |
| **R_pim_** | 0.075 (0.329) | 0.029 (0.360) | 0.1138 (0.6303) |
| **CC_1/2_** | 0.993 (0.760) | 0.999 (0.760) | 0.999 (0.954) |
| **Reflections used in refinement** | 71088 | 12212 | 76000 (7466) |
| **Reflections used for R-free** | 3644 | 562 | 3801 (373) |
| **R_work_** | 0.200 | 0.224 | 0.2081 (0.3626) |
| **R_free_** | 0.248 | 0.264 | 0.2644 (0.4243) |
| **PDB Id** | 8U7J | 8U9E | 8QOC |
| **Number of non-hydrogen atoms** | 39717 | 3468 | 16392 |
| **macromolecules** | 39657 | 3421 | 16217 |
| **ligands** | 60 | 46 | 97 |
| **solvent** | 0 | 1 | 78 |
| **Protein residues** | 5460 | 498 | 2163 |
| **RMS(bonds)** | 0.002 | 0.001 | 0.008 |
| **RMS(angles)** | 0.46 | 0.39 | 1.01 |
| **Ramachandran favored (%)** | 94.10 | 97.55 | 94.17 |
| **Ramachandran allowed (%)** | 5.86 | 2.24 | 5.32 |
| **Ramachandran outliers (%)** | 0.04 | 0.20 | 0.51 |
| **Rotamer outliers (%)** | 0.00 | 0.35 | 1.99 |
| **Clashscore** | 9.17 | 2.89 | 10.47 |
| **Average B-factor** | 67.35 | 93.58 | 62.09 |
| **macromolecules** | 67.37 | 93.53 | 62.20 |
| **ligands** | 53.66 | 97.77 | 53.10 |

Statistics for the highest-resolution shell are shown in parentheses.

**Supplementary Figure S3**. SEC-MALS analysis of SaPdx1 in buffers 1, 2 and 3 in the presence and absence of its substrates. (left panel) SaPdx1 is observed as predominantly hexameric specie in the absence of its substrates. Adding R5P or R5P+ammonia do not change the equilibrium towards the dodecameric species, however, the combination of R5P+ammonia+G3) completely shift the equilibrium towards the active dodecameric assembly. In buffer 2 (inner panel) and buffer 3 (right panel), SaPdx1 is already found in dodecameric specie and adding the substrates does not promote any changes in the oligomeric state of the protein.


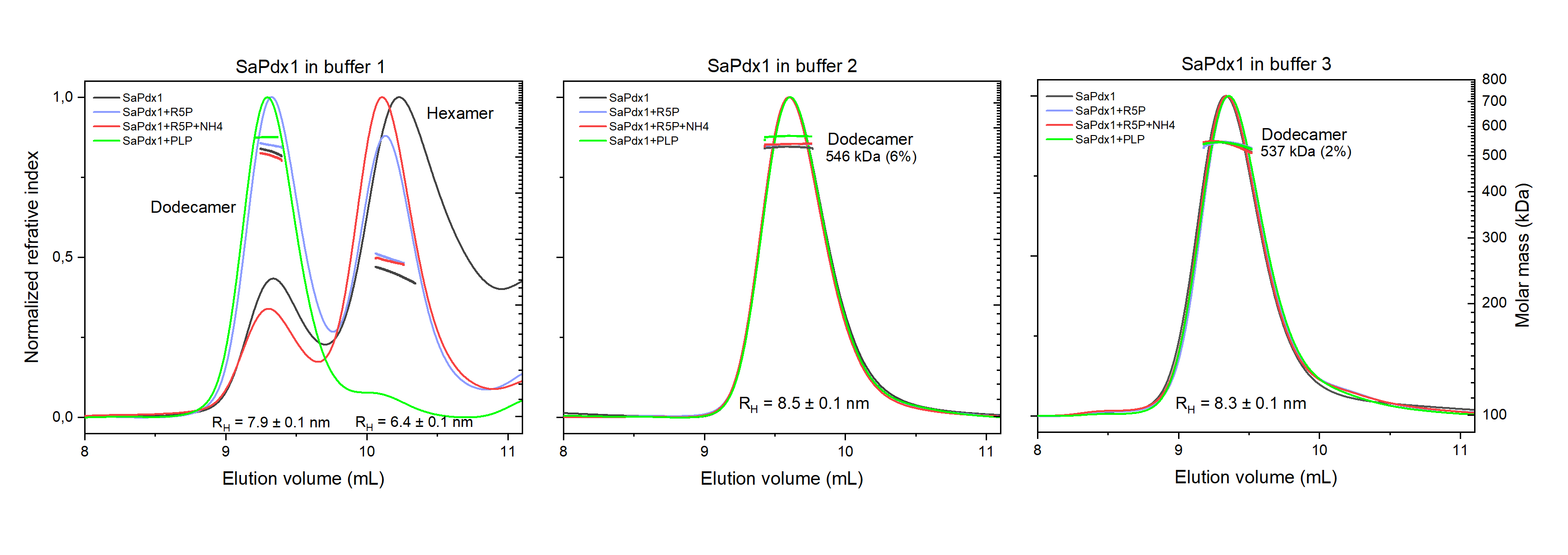


**Supplementary Figure S4**. SEC-SAXS profile for SaPdx1 samples. The sample in buffer 1 was analyzed in batch SAXS experiment and is not shown. Samples in buffers 2 and 3 were analyzed in SEC-SAXS and their profile are shown below, together with the radius of gyration of the frames used in the analysis (right vertical axis).


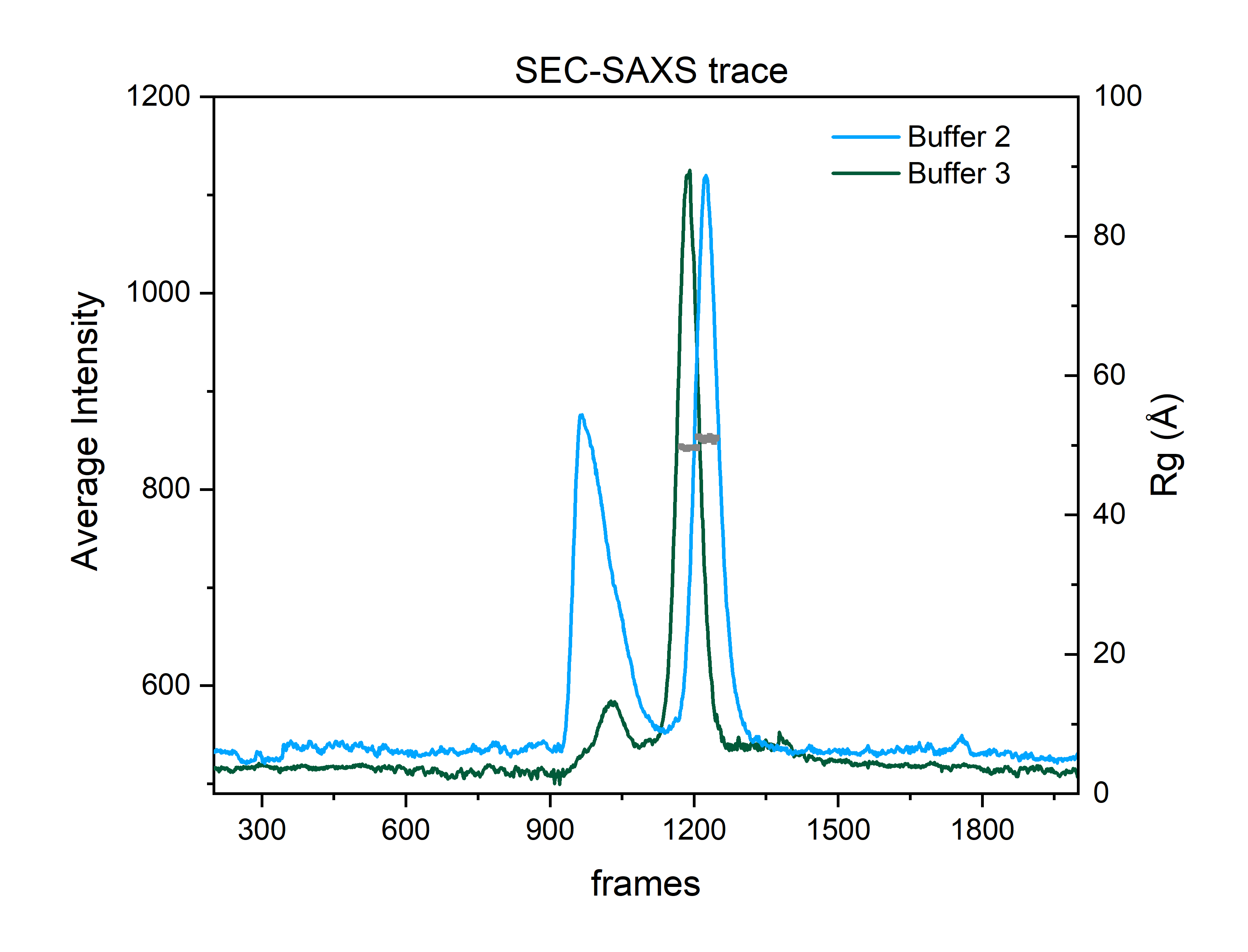


**Supplementary Figure S5**. SEC-SAXS profile for SaPLP synthase complex samples. The SEC-SAXS profile for SaPdx1-2_wt_ and SaPdx1-2_mut_ are shown in cyan and purple lines, respectively, together with the radius of gyration of the frames used in the analysis (right vertical axis). Note the significant difference in the radius of gyration between the samples.


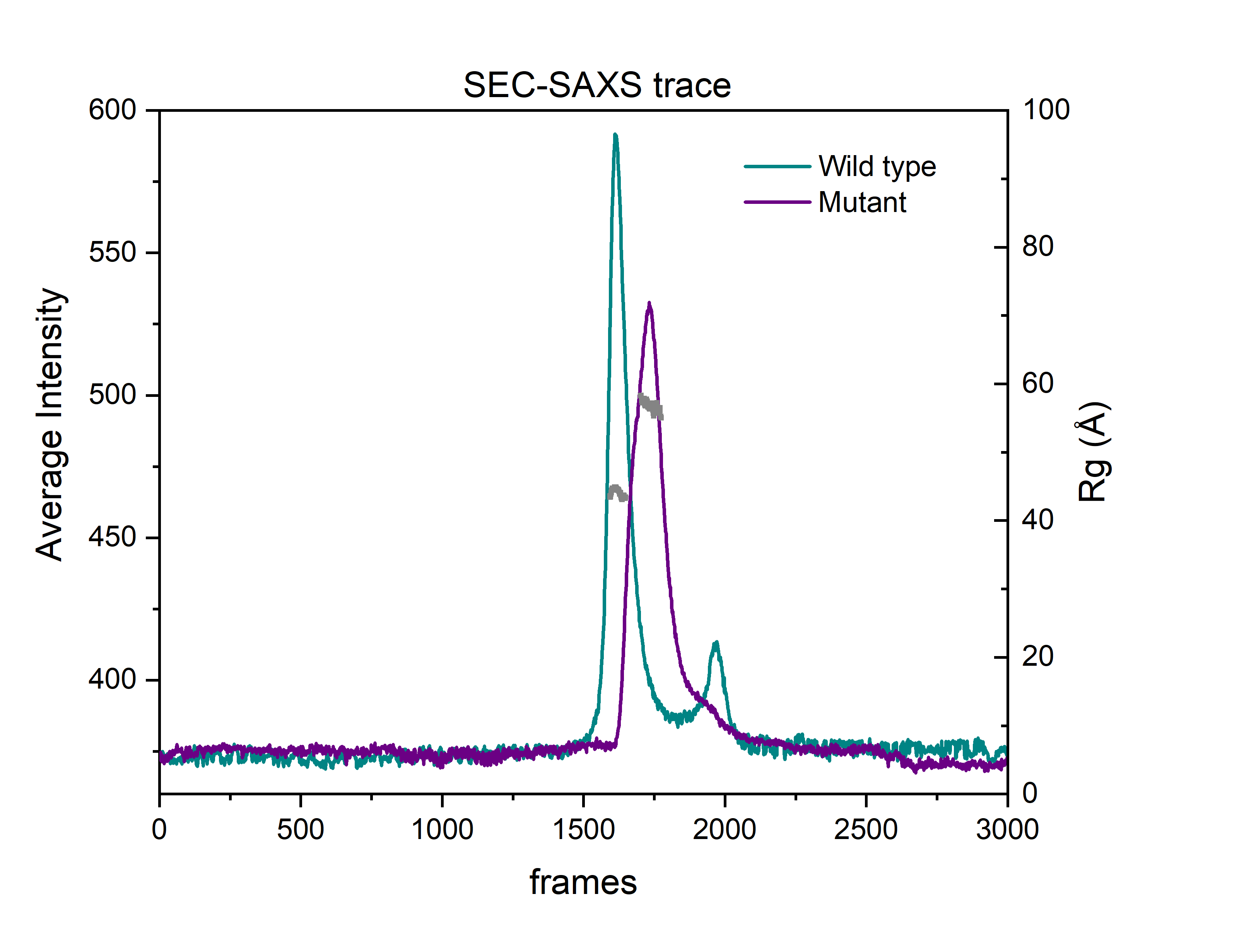

Supplement: Supporting Information [file mmc1.docx]
